# The impact of remote care on the quality of care of pregnant women with diabetes: a systematic review protocol

**DOI:** 10.1186/s13643-026-03244-4

**Published:** 2026-06-23

**Authors:** Sara Sousi, Geva Greenfield, Benedict W. J. Hayhoe, Natasha Singh, Azeem Majeed, Ara Darzi, Ana Luísa Neves

**Affiliations:** 1 Institute of Global Health Innovation, Imperial College London, London, UK; 2 Department of Primary Care and Public Health, Imperial College London, London, UK; 3 Department of Obstetrics & Gynaecology, Chelsea and Westminster Hospital NHS Foundation Trust, London, UK; 4 Department of Metabolism, Digestion and Reproduction, Imperial College, London, UK

**Keywords:** Gestational diabetes mellitus, Diabetes in pregnancy, Remote care, Virtual care, Remote monitoring, MHealth

## Abstract

**Background:**

Diabetes during pregnancy, whether gestational diabetes mellitus (GDM) or pre-existing diabetes mellitus (DM), is associated with increased maternal and neonatal risks. Remote care has shown promise in managing diabetes in the general population, however, its impact on the quality of antenatal care for pregnant women with diabetes remains unclear. Considering the growing adoption of remote care globally, it is vital to understand its impact on the quality of care. This review aims to evaluate the impact of remote care on the quality of care for pregnant women with diabetes. This manuscript describes the protocol for the review.

**Methods:**

MEDLINE, EMBASE, CINAHL, MIDIRS, Scopus, Cochrane, and Global Health databases will be searched to identify studies published from 2005 to 2025. Eligible studies will include original empirical research, encompassing both interventional (e.g. randomised controlled trials) and observational designs (e.g. cohort and cross-sectional studies), assessing remote care, including both virtual consultations and remote monitoring, for pregnant women with GDM, Type 1 or 2 DM. Quality of care will be assessed using the Institute of Medicine healthcare quality framework: patient-centredness, effectiveness, safety, efficiency, timeliness, and equity. Screening and data extraction will be conducted by two independent reviewers. The quality of the studies will be assessed using the Cochrane Risk of Bias tools. Subgroup analyses will be undertaken to explore variation by diabetes and intervention type. This protocol follows the Preferred Reporting Items for Systematic Review and Meta-Analysis Protocol (PRISMA-P) guideline.

**Discussion:**

The findings of this review will provide evidence on the impact of remote care on the quality of care for pregnant women with pre-existing DM and GDM. Strengthening the evidence will support the development of evidence-based implementation strategies and inform policy decisions to ensure safe, effective, and patient-centred use of remote care interventions.

**Systematic review registration:**

PROSPERO CRD420251024685.

**Supplementary Information:**

The online version contains supplementary material available at https://doi.org/10.1186/s13643-026-03244-4.

## Background

Diabetes in pregnancy, either gestational diabetes mellitus (GDM) or pre-existing diabetes mellitus (DM), increases maternal and neonatal risks. GDM affects around 14% of all pregnancies globally, with an increasing prevalence due to risk factors such as obesity and previous pregnancies with GDM [1]. Key risk factors for GDM include advanced maternal age, elevated body mass index (BMI), certain ethnic backgrounds, a history of macrosomic birth (i.e. birth weight > 4 kg), prior GDM, and a family history of diabetes [2, 3]. Given the rising maternal age and the ongoing obesity epidemic, particularly in Western countries, an increasing number of women are at risk of developing GDM or having a pregnancy affected by diabetes, placing both themselves and their babies at heightened risk of adverse health outcomes.

Pregnant women with pre-existing DM are at an increased risk of hypoglycaemia, diabetic ketoacidosis, worsening diabetic retinopathy, diabetic nephropathy, and have higher rates of foetal malformations and miscarriage compared to women without DM [4]. Furthermore, intrauterine exposure to hyperglycaemia increases the risk of T2DM in later life of the baby [5]. All pregnant women with diabetes have an increased risk of pre-eclampsia, premature birth, and caesarean delivery [6]. Neonatal risks include increased risk of birth defects, macrosomia, and hypoglycaemia, whilst women with GDM have an increased risk of developing obesity and/or T2DM later in life and post-natal cardiovascular complications [7, 8].

Diabetes in pregnancy services are increasingly overburdened by the rising numbers of GDM and T2DM pregnancies which are linked with poorer patient experiences and worsening clinical outcomes [9]. Furthermore, although screening for GDM is recommended, universal versus risk factor-based screening often is limited by resource capacity rather than risk profiles [10]. Virtual care platforms and remote monitoring with integration of continuous glucose monitoring have the potential for sustainable, scalable, and patient-centred models that can improve quality of care.

Remote care, including both virtual consultations and remote monitoring, has been increasingly implemented for the management of non-communicable diseases. The Institute of Medicine (IoM) quality of care framework will be used to map the impact on quality of care; this includes patient-centredness, effectiveness, safety, efficiency, timeliness, and equity [11, 12]. Previous systematic reviews have applied this framework to evaluate remote care in primary care and mental health settings [13–15]. The implementation of virtual care can be an effective alternative model for the control of HbA1c in patients with T2DM, particularly in terms of clinical effectiveness, efficiency, patient-centredness, and timeliness. However, mixed results have been reported for safety and equity, with key determinants including lower digital literacy and the reduced opportunity for physical assessments [16, 17].

This systematic review aims to summarise the impact of remote care on the quality of care in the management of pregnant women with diabetes. This manuscript presents the protocol for that review. Specific objectives include (1) to systematically characterise existing initiatives of remote care in this context and (2) to summarise the evidence on how these interventions affect the overall quality of care, using the six domains of healthcare quality as defined by the IoM. 

## Methodology

This protocol has been developed using the Preferred Reporting Items for Systematic Review and Meta-Analysis Protocols (PRISMA-P) [18]. This review has been registered with the International Prospective Register of Systematic Reviews (PROSPERO): CRD420251024685. The systematic review will be reported following the PRISMA 2020 guideline [19].

### Literature search

The electronic databases MEDLINE (via Ovid), EMBASE, Cumulative Index to Nursing and Allied Health Literature (CINAHL), Scopus, Cochrane, Maternity & Infant Care Database (MIDIRS), and Global Health databases will be searched for studies published over the last 20 years (2005–2025). The year 2005 was selected as the start date as it corresponds to the period during which broadband internet access and mobile telephone networks became sufficiently widespread to enable scalable remote care delivery, and coincides with the earliest published evidence of structured telehealth programmes for diabetes management in pregnancy. Whilst telephone-based interventions predate this period, the literature prior to 2005 is sparse, heterogeneous, and largely predates the digital health frameworks used to evaluate contemporary remote care; a scoping exercise confirmed negligible additional yield from extending the search further back. The reference lists of relevant articles (including systematic reviews), grey literature sources (including PROSPERO, reports of relevant stakeholder organisations), and conference proceedings of related conferences will also be searched to identify possible additional studies meeting the inclusion criteria. The search string will include the following themes, and using MeSH and Boolean operators the search strategy will be created; terms will include “telehealth”, “telemedicine”, “remote care”, “virtual wards”, “gestational diabetes”, and “diabetes in pregnancy”. These examples are illustrative; the complete search strategy, including all MeSH terms and Boolean operators, is provided in Supplementary Table 1. The search strategy was developed in collaboration with, and optimised by, an experienced librarian; an example is presented in Supplementary Table 1.

### Study selection criteria

The Population, Intervention, Comparison, Outcomes, and Study Design (PICOS) framework was used to define inclusion and inclusion criteria. Studies will be included if they have a population of pregnant women with a definitive diagnosis of diabetes, including GDM, T1DM, or T2DM. No restrictions will be placed on the timing of diabetes screening during pregnancy, provided a definitive diagnosis is established.

Studies evaluating the use of remote care solutions for the management of diabetes during pregnancy will be included. Studies comparing remote care interventions to traditional in-person clinical care will be included to assess the impact of remote care on quality of care compared with standard practice. Studies focusing on remote care or digital interventions for the management of diabetes in non-pregnant individuals, or those utilising digital tools solely for education or psychological support, will be excluded.

The primary outcomes will focus on the six IoM domains of healthcare quality: effectiveness, safety, efficacy, timeliness, equity, and patient-centredness. Study-reported outcomes (e.g. glucose control, maternal outcomes, neonatal outcomes, healthcare utilisation, and patient-reported measures) will be extracted and subsequently mapped to the relevant IoM domains to enable structured synthesis across these quality dimensions. Where multiple outcome measures are reported within a domain, all relevant measures will be captured to minimise outcome reporting bias. Secondary outcomes will include additional study-specific indicators related to care delivery or implementation (e.g. service utilisation, adherence to monitoring protocols, or resource use) that do not map directly to the IoM framework but provide contextual information regarding the delivery of remote care interventions.

Original research articles, including experimental studies, controlled trials, and observational studies, will be included; whilst reviews, abstracts, case reports, non-full-text articles, and study protocols will be excluded. Purely qualitative studies will be excluded from the primary synthesis because the review is designed to quantify impact across the six IoM domains and to enable meta-analysis where feasible. However, we recognise that excluding qualitative evidence may limit insight into patient-centredness and equity, which are inherently experiential domains and less amenable to quantitative measurement. To address this, if a sufficient body of relevant qualitative studies is identified during screening, these will be analysed separately through a parallel qualitative synthesis to complement the quantitative findings. There will be no restrictions based on country or language. The full PICOS-based inclusion and exclusion criteria are defined in Table 1.

**Table 1:** Inclusion and exclusion criteria of the Population, Intervention, Comparison, Outcomes, and Study (PICOS) framework

	Inclusion criteria	Exclusion criteria
Population	Pregnant women with pre-existing diabetes mellitus (type 1 or type 2) Pregnant women with gestational diabetes mellitus	Non-pregnant women with diabetes mellitus (type 1 or type 2) Children (age < 16) or men with diabetes mellitus (type 1 or type 2) Post-partum women
Intervention	Remote care for clinical management of diabetes in pregnancy, this can include: - Virtual/remote consultations (telephone, video, online, chat-based) - Remote monitoring applications (such as CGMs) - Virtual wards	Teletherapy support interventions Virtual reality interventions Digital tools aimed for post-pregnancy Digital tools aiding in diagnosis and/or screening of pregnant women for gestational diabetes mellitus Digital interventions that are lifestyle, exercise, and education purposed
Comparator	Face-to-face monitoring Usual standard of care	N/A
Outcomes	Institute of Medicine quality of care domains, which encompass: - Effectiveness - Safety - Efficiency - Timeliness - Equity - Patient-centredness	N/A
Study type	Studies with empirical data, original research: randomised control trials, non-randomised control trials, cohort studies, retrospective studies, cross-sectional studies, case–control studies	Reviews, abstracts, editorials, book chapters, commentaries, case reports, non-full-text studies, protocols, conference proceedings

### Study screening and data extraction

The studies yielded from the selected databases will be screened using Covidence, a web-based systematic review management software. The studies will be screened by two reviewers independently. This will include title and abstract screening followed by full-text screening based on the pre-defined inclusion and exclusion criteria (Table 1). Any discrepancies will be solved by consensus and/or a third independent reviewer. Interrater reliability will be reported using Cohen’s kappa [20].

Data extraction will be undertaken independently by two reviewers into a pre-populated standardised extraction template (Supplementary Table 2). Study information, including title, authors, publication year, study type, population characteristics and size, diabetes type, intervention type and details (type, duration, frequency), setting, clinical outcomes, and maternal and neonatal outcomes, will be extracted. Any discrepancies in the data extracted will be resolved by consensus.

### Study quality

The Cochrane Risk of Bias 2 (RoB2) tool will be used for randomised control trials, and the Risk Of Bias In Non-randomised Studies of Interventions (ROBINS-I) tool for non-randomised [21, 22]. The studies will be scored by two independent reviewers, with any discrepancies being resolved via consensus or a third independent reviewer. The results will be reported using traffic light plots showing each risk of bias domain for each included study.

### Data analysis

Initially a narrative synthesis will be undertaken; it will include the studies’ characteristics and findings. Summaries will be provided by intervention and diabetes type, and across the six healthcare domains defined by the IoM for assessing quality of care. Tables will be used to summarise the extracted patient safety indicators and study descriptives, including population demographics, intervention type, and outcomes.

A meta-analysis will be performed if there are sufficient studies with a comparator meeting the inclusion criteria. To minimise bias arising from pooling heterogeneous study designs, randomised controlled trials and non-randomised studies (including cohort, cross-sectional, and retrospective designs) will be analysed separately. For the outcomes of interest, effect measures will be derived from reported incidence rates or mean values, as appropriate. The meta-analysis will focus on evaluating the measurable impact of the digital interventions on quality of care, therefore providing more robust evidence around the feasibility, scalability, benefits, and potential risks in antenatal care pathways. Heterogeneity will be assessed using the *I*^2^ statistic, which will be classified as low (0 to 25%), moderate (25 to 75%), or high (above 75%). Depending on the degree of heterogeneity, either a fixed-effects or random-effects model will be used. Outcomes will be presented using forest plots, including measures of heterogeneity and *p* values for overall effect.

Subgroup analyses will be conducted based on diabetes type (GDM, T1DM, T2DM) and intervention type (e.g. remote app monitoring versus teleconsultation), contingent on the characteristics of the included studies. Given that not all studies are expected to include both gestational and pre-existing diabetes, subgroup analyses will be performed for assessing variations in the impact of intervention type and diabetes type on quality of care. In addition, based on study risk of bias (high versus low risk), subgroup analyses may be performed to explore potential sources of heterogeneity. Given that a number of interventions are likely to be multi-component in nature, making it difficult to isolate the contribution of remote care alone, a sensitivity analysis will be conducted restricted to studies in which remote care is the sole or primary intervention component, in order to examine the independent impact of remote care on quality of care outcomes. Publication bias will be assessed using funnel plots, and statistically through Egger’s test.

The handling of missing data will be addressed at both the study and outcome levels. Where outcome data are missing or incompletely reported, the study authors will be contacted where feasible. Where contact is not possible or unsuccessful, studies with missing data will be narratively described rather than included in the meta-analysis. The extent of missing data will be documented transparently in the results, and sensitivity analyses will be undertaken to assess the potential impact of missing data on the pooled estimates where appropriate.

Analyses will be performed using R and Microsoft Excel.

## Discussion

Diabetes in pregnancy is increasing globally, with well-established maternal and neonatal risks, effective management, especially of GDM, can improve outcomes and reduce long-term disease burden and costs [23–25]. This systematic literature review aims to bring together the existing evidence about the role of remote care in managing pregnant women with diabetes.

### Strength and limitations

This systematic review’s protocol has been developed in accordance with the PRISMA-P framework. This review will provide an understanding of the current landscape of remote care within the antenatal pathway for pregnant women with diabetes, evaluate its impact on outcomes compared to standard in-person care, and identify areas across the six IoM domains of healthcare quality where remote care is effective and where further improvement is needed. The search strategy was designed and optimised in collaboration with an experienced librarian, and multiple databases will be systematically searched. No language or country restrictions will be applied, enhancing the global relevance of the findings. Outcomes have been aligned with IoM domains of healthcare quality, providing a structured and internationally recognised framework for evaluating remote care for pregnant women with diabetes. A framework has been previously used to assess the impact of remote care on specific disease populations [13, 14].

There are several potential limitations to be considered. Firstly, the amount and heterogeneity of the available evidence may pose challenges for synthesis, particularly given the variation in the types of remote care interventions assessed. Remote care encompasses a range of interventions, including telephone and video consultations, app-based interactions, and remote monitoring via connected devices. This introduces variability and limits the ability to make direct, like-for-like comparisons across studies. In addition, many interventions may be part of a multi-component approach, which would make it difficult to assess the impact on quality of care due to the remote care alone. Secondly, heterogeneity in the methods used to collect and report outcomes across studies may limit the ability to conduct a robust meta-analysis and draw definitive conclusions. Thirdly, a wide range of digital and telemedicine interventions are included necessitating subgroup analyses, as these interventions are not directly comparable and cannot be meaningfully aggregated. Thus, a potential limitation is that there may be an insufficient number of studies per each intervention type to support robust subgroup analyses.

### Implications and expected study impact

Given the rising prevalence of diabetes in pregnancy, synthesising the current evidence on remote care is both timely and essential. Whilst there are already published systematic reviews on the effectiveness and safety of telemedicine interventions, they do not capture all six domains of healthcare quality [26–29]. Existing reviews have focused predominantly on glycaemic control and selected pregnancy outcomes, with Huang et al. and Xie et al. restricted to RCTs comparing telemedicine to standard care in GDM, and Wang et al. and Hangaard et al. similarly omitting dimensions such as efficiency, timeliness, patient-centredness, and equity from their synthesis. None has applied a structured quality-of-care framework to evaluate remote care holistically across the antenatal context. This systematic review will synthesise the evidence on how these interventions affect the overall quality of care across the six domains of healthcare quality as defined by the IoM: patient-centredness, effectiveness, safety, efficiency, timeliness, and equity. This structured framework will clarify the benefits and potential risks associated with remote models of care in the antenatal context and identify key clinical and patient safety indicators.

Our findings will have implications for research, policy setting, and clinical practice. In research, the review will identify evidence gaps and help define priorities for future studies, including the comparative effectiveness of remote versus in-person antenatal care models. In policy, it will support evidence-based policymaking for the implementation of remote care in antenatal services. In clinical practice, it will help to guide healthcare professionals on the safe and effective integration of remote care models into standard care pathways.

Additionally, the review will explore whether certain subgroups, such as women with GDM or T1DM or T2DM, experience different outcomes under remote care. Understanding subgroup variations could facilitate the development of more tailored and equitable remote care pathways. By mapping impacts across the six domains of healthcare quality, the review will also highlight areas where remote care models may need further optimisation, particularly in terms of safety and equity. Ultimately, we anticipate that findings of this review will support the safe, effective, and scalable implementation of remote care models to improve outcomes for all pregnant women with diabetes.

## Supplementary Information

Supplementary Material 1.

## Data Availability

Not applicable.

## Abbreviations

BMI: Body mass index
CINAHL: Cumulative Index to Nursing and Allied Health Literature
DM: Diabetes mellitus
GDM: Gestational diabetes mellitus
IoM: Institute of Medicine
MIDIRS: Maternity & Infant Care Database
PICOS: Population, Intervention, Comparison, Outcomes, and Studies
PRISMA: Preferred Reporting Items for Systematic Review and Meta-Analysis
PRISMA-P: Preferred Reporting Items for Systematic Review and Meta-Analysis Protocols
PROSPERO: Prospective Register of Systematic Reviews
RoB2: Risk of Bias 2
ROBINS-I: Risk Of Bias In Non-randomised Studies of Interventions
T1DM: Type 1 diabetes mellitus
T2DM: Type 2 diabetes mellitus
