# Supplementary material for: The impact of remote care on the quality of care of pregnant women with diabetes: a systematic review protocol
Source: Syst Rev. 2026 Jun 23;15:242. doi: 10.1186/s13643-026-03244-4 (PMC13543511; doi:10.1186/s13643-026-03244-4)
Supplement: Supplementary file 1 [file 13643_2026_3244_MOESM1_ESM.docx]

**Supplementary Materials**

**Supplementary Table 1:** Example search string of MEDLINE database via Ovid

| **Theme** | **#** | **Query** | **Results from Apr 25, 2025** |
| --- | --- | --- | --- |
| **Remote Care** | **1** | exp Telemedicine/ or telemedicine.mp. | 65,654 |
|  | **2** | exp Remote Consultation/ | 6,151 |
|  | **3** | ((online or phone* or remote* or tele* or video* or virtual*) adj2 (appointment* or care or consult* or hospital* or monitor* or visit* or ward*)).mp. | 47,114 |
|  | **4** | (digital health or dhealth or d-health or home monitor* or mobile health or mHealth or m-Health or smartphone* app* or mobile* or long distance consultation* or teleconsult* or tele-consult* or telemonitor* or tele-monitor* or telehealth* or tele health* or tele-health* or telemetry or tele-homecare or telehomecare or remote surveillance or remotely delivered or tele nursing or telenursing).mp. | 215,743 |
|  | **5** | 1 or 2 or 3 or 4 | 275,809 |
| **Pregnancies with Diabetes** | **6** | exp Diabetes, Gestational/ or gestational diabetes.mp. | 29,703 |
|  | **7** | exp Pregnancy/ and exp Diabetes Mellitus/ | 30,731 |
|  | **8** | (pregnan* adj3 (gly* or diabet* or DM or GDM)).mp. | 23,066 |
|  | **9** | (pregestational diabetes or pre-gestational diabet* or maternal diabet*).mp. | 3,829 |
|  | **10** | exp Pregnancy in Diabetics/ | 14,224 |
|  | **11** | 6 or 7 or 8 or 9 or 10 | 48,911 |
| **Patient-centredness** | **12** | (((experience* or satisfaction) adj4 (patient* or consumer* or client* or survey* or questionnaire*)) or PREM* or patient-reported experience measure* or patient-cent?red* or person-cent?red*).mp. | 885,044 |
|  | **13** | exp Patient Satisfaction/ | 106,148 |
|  | **14** | 12 or 13 | 894,314 |
| **Effectiveness** | **15** | exp Treatment Outcome/ or ((health or clinical* or treatment*) adj3 (outcome* or effective* or efficacy)).mp. | 2,209,106 |
| **Safety** | **16** | ((patient adj3 (safety or harm)) or misdiagnos* or safety manag* or (accident* adj2 prevent*) or error* or medication reconcil* or near miss*).mp. | 642,096 |
|  | **17** | exp patient harm/ or exp patient safety/ or exp Diagnostic Errors/ | 152,256 |
|  | **18** | 16 or 17 | 720,927 |
| **Efficiency** | **19** | (efficien* or economic* or cost* or expenditure* or charge* or (number adj3 appointment*) or (number adj3 admission*) or (number adj3 consultation*)).mp. | 2,555,630 |
| **Timeliness** | **20** | (wait* list* or wait* time* or timeliness).mp. | 50,297 |
|  | **21** | exp Time-to-Treatment/ or exp Waiting Lists/ | 25,773 |
|  | **22** | 20 or 21 | 60,782 |
| **Equity** | **23** | ((health* or health care or access) adj3 (equity or disparit* or inequit* or inequalit* or equality or gap)).mp. | 114,822 |
|  | **24** | exp Health Equity/ | 5,151 |
|  | **25** | 23 or 24 | 114,822 |
| **IoM Domains** | **26** | 14 or 15 or 18 or 19 or 22 or 25 | 5,964,615 |
|  | **27** | 5 and 11 and 26 | 237 |
|  | **28** | limit 27 to yr="2005 -Current" | 226 |

**Supplementary Table 2:** Standardised Data Collection Template

| Author |  |
| --- | --- |
| Publication Year |  |
| Title |  |
| Journal |  |
| Country |  |
| Setting |  |
| Study type |  |
| Study aim |  |
| Sample size (n (i; c)) |  |
| Demographic characteristics |  |
| Intervention type |  |
| Date of intervention |  |
| Follow-up period |  |
| Participants - demographics |  |
| Diabetes type - T1DM, T2DM, GDM (n (i; c)) |  |
| Source of data |  |
| Study design and comparison |  |
| Inclusion Criteria |  |
| Exclusion Criteria |  |
| Retention or adherence rate (n (i; c)) |  |
| Response rate (n (i; c)) |  |
| Type of technology |  |
| Outcomes assessed |  |
| Key results (mean difference, OR) |  |
| Adjustment for confounders |  |
| Other key findings |  |
| Domain(s) of quality |  |
| Method of recruitment |  |
| Source of funding |  |
| Possible conflicts of interest |  |
